# Editorial

**Published:** 1872-08

**Authors:** 


### EDITORIAL.

### Thymol.

The question is often asked "What about thymol?" This agent has been before the profession for about two years, but few however having tested it, or introduced it into practice; a few have used it constantly since its introduction.

There is some difference of opinion in reference to its value; some claiming that it is as good if not better than carbolic acid in every respect. In this some take exceptions, avering that for exposed pulps it is objectionable, as in almost all cases severe pain results from its application. This it is claimed by some is caused by the glycerine which is used as a solvent and diluent for the thymic acid. This is a matter easily decided by experiment, which everyone should make for himself. The fact is undeniable however that in most cases, nearly all indeed, severe pains follows its use on pulps. Of this class of agents for treating this tissue there is perhaps nothing better than carbolic acid; at least our experience thus far warrants such a conclusion. In the treatment of sensitive dentine thymol is quite as efficient as corbolic acid or anything we have used; and in the treatment of pulpless teeth, for correcting the fetid and offensive condition of pulp chambers and canals, in which the tissue has died and decomposed, it is better than anything we have ever used. It acts promptly as a deodorizer, and the disinfection is permanent, unless new debris is developed. It is a good stimulant, but does not possess the escharotic property to the some extent as carbolic acid, it does not therefore combine as readily with any constituent of the living tissue. In the treatment of alveolar abscess in some cases it serves a valuable purpose, while in others it is of no value whatever, owing, in part at least, to the absence of more vigorous scharotic properties. We do not regard "glycerole of thymol" as the best therapeutic agent in the treatment of exposed pulps; but it is good in the treatment of sensitive dentine, and very good in the treatment of pulpless teeth, and of some value in the treatment of alveolar abscess, only however in cases of the milder form, and in connection with good constitutions. In the use of this agent, as with all others, a thorough knowledge, and an active discriminating judgment should govern.

### American Dental Association.

The annual meeting of this body has just been held at Niagara Falls, and in many respects it was the most successful meeting of this association ever held. A larger number was in attendance than ever before, and the character of the proceedings was certainly an improvement on that of former times. In regard to the organization some steps were initiated that will remedy many defects, bring order where there was confusion, and make its working more smooth and harmonious; of them in detail, we shall have more to say hereafter.

Nearly all the committees presented and read reports upon their respective subjects. The discussions upon these were very interesting and instructive. As usual two mnch time was occupied by irregular miscellaneous business; usually in such matters, unfortunately, every body wishes to put a finger, and sometimes stick in two or three; in the future we apprehend this will become less.

Four days and evenings were occupied by the sessions. Clinics were held two or three times and were very interesting and perhaps profitable, to about a dozen each time; and by the rest, pleasant social and professional intercourse were enjoyed, and possibly not the least profitable of the exercises.

A great many new instruments and appliances were on exhibition some of which doubtless are valuable. We shall notice some of these at another time.

Some important subjects involving the foundation principles of the profession were overlooked or only referred to in a casual way. We think far more time and attention should be given to some of the great interests of the profession that are usually ignored.

The next meeting will be held at Put-in-Bay, and on many accounts no better place could have been selected, and we doubt not it will be to the eminent satisfaction of all who shall attend in 1873. And we trust that a larger meeting shall then convene than ever before.

### What Shall be done with Josiah?

The above seems to be the general query just now in the dental profession. Well, if we were going to answer the question again we should say, *Let him alone, and let the rubber alone.* This is about the only course any one can pursue and maintain his self respect, and avoid being swindled, and keep himself free from a course of practice that has occasioned more mischief, produced more demoralization, and thwarted to a greater extent the efforts of those who have labored most assiduously for the advancement of the profession than anything that has ever occurred.

Indeed the use of rubber in dental practice as a base for artificial teeth has been an unmitigated curse from the beginning to the end, damaging to both the dentist and his patient. But the profession, seems for the most part, to be in about the same condition of the man who had the bear by the paws around a tree, it was "pretty hard work to hold on, and dangerous to let go." Now let the bear go, and if he comes around the tree fight him. Nothing has ever demoralized the dental profession like the use of hard rubber. It was a means of introducing into the profession a host of incompetents and empirics, who soon after rubber was introduced swarmed through the country, worse than the army worm or the potato bug. They not only entertained their own low and depraved notions of the importance of the teeth, but they instilled these false views into the minds of the people; educated them backward; from which errors it will require more than one generation to free the people, and especially will this be difficult when so many, in other respects good men, in the profession concur in, and practically, help to carry forward this demoralization, using that which they know is not fit to be used, and yet putting it off upon their patients as all right, and without one word of protest. The occasion for artificial teeth with the most favorable dentures that are or can be made is and ever should be regarded as a great calamity. Every one should be brought to a realization of this truth by the proper education—not by destroying the natural organs, but by instilling just notions of the importance and value of the natural teeth in all respects, and giving correct views as to what is requisite for their preservation.

Should the artificial limb-maker start out over the country, and induce people to have their lame legs and crippled arms all cut off, assuring them that he could make far better ones—legs that would never ache or get sore, and feet that would never have corns, assuring them that he could make far better ones, and so nice, far better than these old rough scruffy legs, and so very cheap too, only $10 for a leg, and for both legs $15. What! no body would hear him? Yes, there are many who would go one leg on it, and some two, especially if they were somewhat crooked and unsightly, and the new ones were cheap.

Now this would be just as reasonable as the course dental practice has taken with a certain class of dentists upon and since the introduction of hard rubber. Unfortunately the dentists meet with far larger success than the leg man would. Now in view of all the difficulties that attach to the use of hard rubber for artificial dentures, we can not suggest less than its total and immediate abandonment.

